# Challenging Tumor Heterogeneity with HER2, p16 and Somatostatin Receptor 2 Expression in a Case of EBV-Associated Lymphoepithelial Carcinoma of the Salivary Gland

**DOI:** 10.1007/s12105-023-01592-4

**Published:** 2023-10-17

**Authors:** Arlind Adili, Tracy O`Connor, Philipp Wales, Marcus Seemann, Sylvia Höller, Barbara Hummer, Sandra N. Freiberger, Stephan Rauthe, Niels J. Rupp

**Affiliations:** 1 Institute of Pathology, Viollier AG, Allschwil, Switzerland; 2 Department of Biology, North Park University, 3225 W. Foster Avenue, Chicago Illinois, 60625 USA; 3 Hals-, Nasen-, Ohrenmedizin, Kantonsspital Olten, Olten, Switzerland; 4 Radiologie Bürgerspital Solothurn, 4500 Solothurn, Switzerland; 5 Institute of Pathology, Stadtspital Triemli, Zurich, Switzerland; 6 Department of Pathology and Molecular Pathology, University Hospital Zurich, Schmelzbergstrasse 12, 8091 Zurich, Switzerland; 7 Faculty of Medicine, University of Zurich, Zurich, Switzerland

**Keywords:** Salivary gland, Lymphoepithelial carcinoma, EBV, SSTR2, p16, HER2

## Abstract

**Background:**

Lymphoepithelial carcinoma of the salivary glands (LECSG) is a rare disease in the Western hemisphere that is typically associated with an EBV infection. The molecular mechanisms of LECSG tumorigenesis are poorly understood.

**Results:**

Here we report a case of EBV-associated LECSG with an unusual immunophenotype. The tumor exhibited bi-morphic histological features with a mutually exclusive expression of HER2 and p16. The p16-positive domain of the tumor immunohistochemically co-expressed late membrane protein 1 (LMP-1), while the HER2 positive domain did not. Both tumor regions expressed SSTR2.

**Methods:**

In situ hybridization confirmed the EBV origin of the tumor while extensive immunohistochemical characterization and the recently established RNA-based next generation sequencing panel (“SalvGlandDx” panel) did not reveal evidence for another salivary gland neoplasm. No HPV co-infection was detected by in situ hybridization or PCR-based screenings and no ERBB2 gene amplification was detected by fluorescence in situ hybridization.

**Conclusion:**

These findings suggest tumor heterogeneity and lack of genomic aberrations in EBV-associated LECSGs. The heterogenous and unusual immunohistochemical features explain the diagnostic difficulties and simultaneously extend the immunophenotype spectrum of this tumor entity.

## Introduction

Lymphoepithelial carcinoma of the salivary glands (LECSG) is common in Epstein-Barr virus (EBV) endemic areas such as Southeastern Asia and the Arctic and accounts for up to 90% of malignant salivary gland tumors in these regions [1], [2]. LECSG is typically found in the parotid gland of patients in their 5th decade and occurs with equal frequency in both sexes [1, 3]. Surgical resection often combined with radiation therapy is the preferred treatment modality, and the overall 5-year survival rate is > 80% [3].

Histologically, LECSG is characterized by syncytial growth of undifferentiated epithelial cells, usually accompanied by prominent lymphoplasmacytic infiltration [4]. Immunohistochemically, LECSG is typically positive for pancytokeratin, CK5/6 as well as the squamous epithelial markers p40 and p63 [3], [4]. Apart from the close association of LECSG with EBV infection, there are no other established specific immunohistochemical or molecular markers for this malignancy. The exact molecular mechanisms of LESCG tumorigenesis remain poorly defined. Similar to the more common EBV-associated nasopharyngeal carcinoma (NPC), LESCG is linked to the expression of the EBV-type 2 latency gene late membrane protein 1 (LMP-1) [5]. In NPC tumorigenesis, a close association of LMP-1 with human epidermal growth factor receptor 1 (HER1) and somatostatin receptor 2 (SSTR2) has been identified [6]. While a few NPC cases with an EBV and HPV co-infection have been described, this has not been the case with LESCG [7], [8], [9, 10]. Likewise, p16 (CDKN2A; p16^INK4a^), the surrogate marker of high-risk HPV association, is typically negative in LESCG [3].

Here we report an unusual case of EBV-associated LESCG in a 72-year-old white European male that expressed SSTR2. Moreover, the tumor exhibited mutually exclusive overexpression of human epidermal growth factor receptor 2 (HER2), another ErbB family receptor tyrosine kinase, and p16.

## Materials and Methods

Immunohistochemical analyses were performed on formalin-fixed and paraffin-embedded (FFPE) sections. Epitope retrieval was conducted according to the manufacturer’s guidelines. The following antibodies were utilized for staining: Androgen receptor (Dako AR441; 1:200), CD117 (Dako A4502; 1:50), DOG-1 (Leica K9; 1:400), HER2 (Cell Marque SP3; 1:80), LMP-1 (Dako M0897; 1:400), ENBA2 (Leica PE2 1:100), CK5/6 (Dako D5/16B4; 1:200), p63 (Dako 4A4; 1:600), p40 (Biocare ACI 3030B; 1:50), p16 (Roche E6H4; 1:2), p53 (Leica DO7; 1:100), Rb1 (Leica 13A10; 1:50), SSTR2 (Abcam UMB1; 1:200).

To detect the non-coding EBV small RNAs, Epstein-Barr Encoding Region (EBER) in situ hybridization was performed on 4-µm thick FFPE samples using the Bond EBER probe (PB0589), anti-Fluorescein antibody (AR0222) and Bond Refine Red Detection kit (DS9390) on a Leica Bond automated staining system according to the manufacturer`s protocol (Leica biosystems).

In situ hybridization for HPV was performed using the RNAscope™ 2.5 VS Probe-HPV-HR18 (No: 312598) and Bond RNAscope Brown Detection Kit (DS9815) on a Leica Bond automated staining system according to the manufacturer`s protocol (Advanced Cell Diagnostics). Screening for E6/E7 mRNA of the HPV high-risk genotypes 16, 18, 26, 31, 33, 35, 39, 45, 51, 52, 53, 56, 58, 59, 66, 68, 73 and 82 was performed.

For HPV screening, DNA was isolated from FFPE blocks utilizing the Promega Maxwell^®^ RSC DNA FFPE Kit. The Allplex™ HPV28 Detection kit (Seegene) including positive controls were used to test for 19 high-risk HPV genotypes (16, 18, 26, 31, 33, 35, 39, 45, 51, 52, 53, 56, 58, 59, 66, 68, 69, 73, 82) and 9 low-risk HPV genotypes (6, 11, 40, 42, 43, 44, 54, 61, 70). Amplification and detection were performed on a CFX 96 real-time PCR detection system (BioRad) and a Ct value of **≤** 43 was considered a positive result.

Fluorescence in situ hybridization using the FISH HER2 staining kit (TA9217; Vysis/Abbott), which includes fluorescently labeled DNA probes covering the HER2 locus (SpectrumGreen; 17q11.2-q12) and a chromosome enumeration probe (CEP) recognizing the centromeric region of chromosome 17 (SpectrumAqua;17p11.1-q11.1). Deparaffinization, pretreatment and proteinase digestion were performed on the Leica Bond slide-staining system, and samples were incubated for 18 h at 37 °C. The fluorescent signals on at least 25 cell nuclei were first independently analyzed by 2 trained lab technicians and were later independently validated by 2 trained pathologists. Samples with a HER2/CEP17 ratio ≥ 2 or HER2/cell ratio ≥ 6 were considered positive for HER2 amplification (Wolff et al., 2018).

Nucleic acid isolation, library preparation, next generation sequencing, and data analyses for the SalvGlandDx panel were performed as described in [11].

## Results

**Fig. 1:**
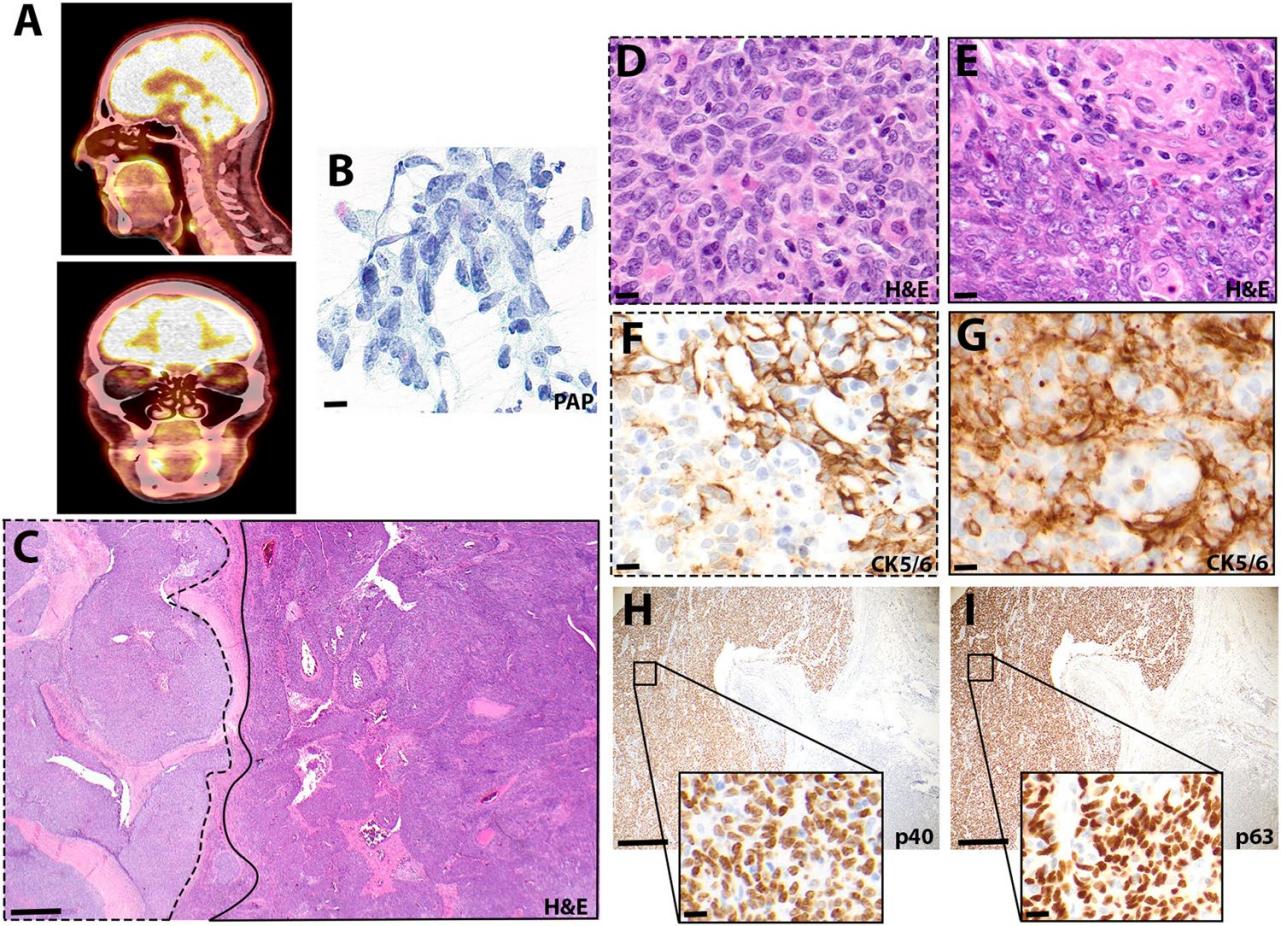
A solitary tumor mass in the parotid gland. **A** ^18^F-FDG-PET computer tomography scan showed no evidence of other tumor manifestations in the head and neck area. **B** A fine needle aspirate showed basaloid cells with high-grade atypia in the Papanicolaou stain, scale bar 20 μm. **C** A representative hematoxylin eosin stain of the lymphoepithelial carcinoma with bi-morphic histological features of the tumor, scale bar 500 μm. A higher magnification of the tumor area with broad sheets of syncytial tumor cells with only few intermingled lymphocytes in **D** and anastomosing tumor islands creating a jigsaw puzzle-like appearance with lymphoid infiltrates in **E**, scale bar 20 μm. Varying expression patterns of CK 5/6 expression within the two tumor regions (**F**) and (**G)**, scale bar 20 μm. The broad sheets of syncytial tumor cells reveal strong nuclear expressions of p40 and p63 and scattered sparse p63 positive nuclei in the jigsaw puzzle-like area in **H** and **J**, scale bar 200 μm, inset 20 μm

A 72-year-old white European male patient presented with a painless and slowly growing swelling in the parotid gland. The initial sonography revealed a 19 × 19 mm circumscribed, hypoechoic and inhomogeneous intraparotid mass. The postoperative 18-fluorodeoxyglucose positron emission tomography/computed tomography (^18^F-FDG PET/CT) scan revealed no additional tumor masses in the remaining head and neck area or in the upper digestive tract, including the cervical lymph nodes (Fig. 1A). The fine needle aspiration yielded an inconclusive diagnosis of basaloid, partly polygonal cells with high-grade nuclear atypia (Fig. 1B), following complete surgical excision. Eight months thereafter the patient remained disease-free.

**Fig. 2:**
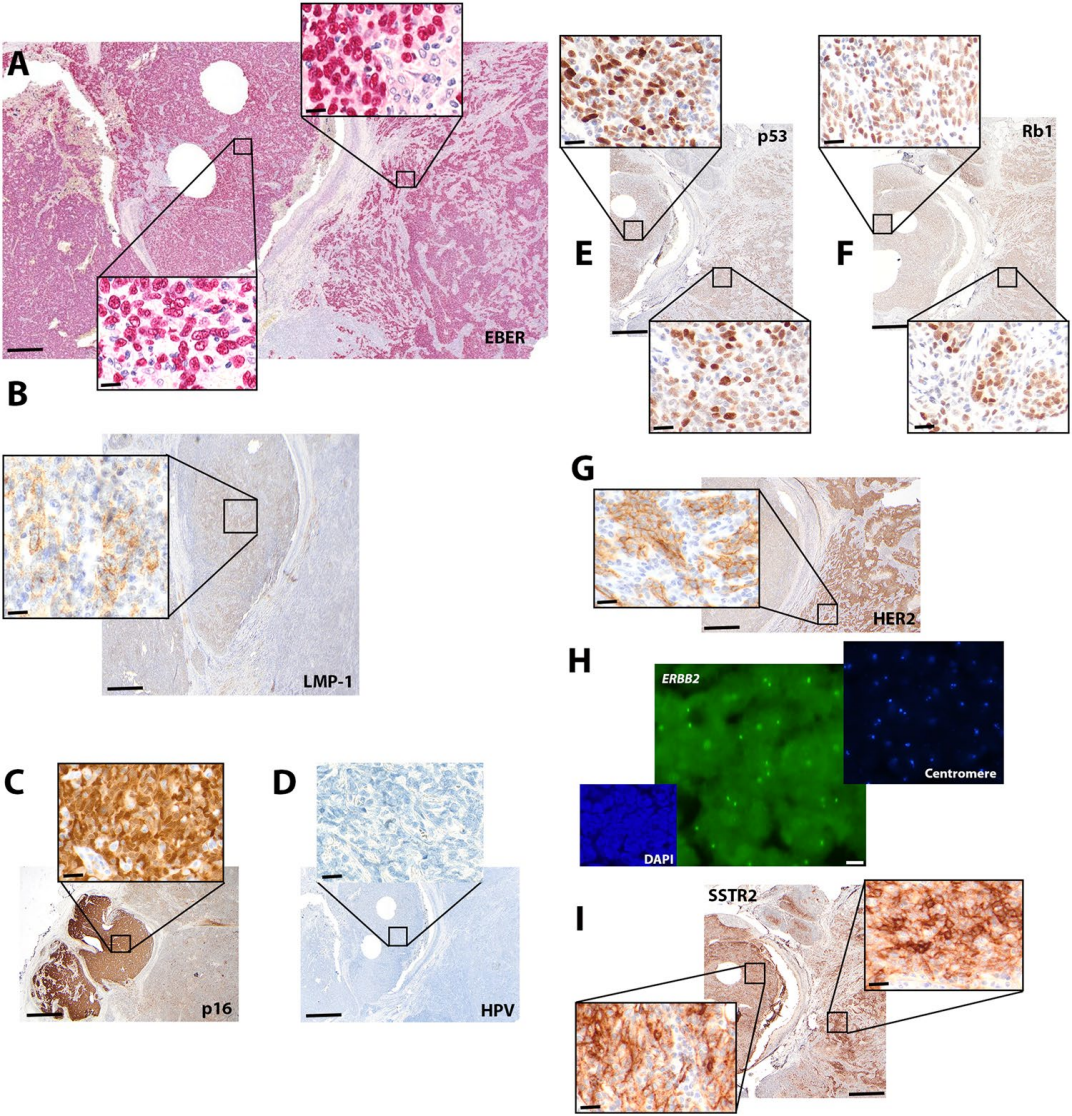
A bi-morphic tumor with SSTR2 and mutually exclusive p16 and HER2 expression. **A** In situ hybridization for EBER confirmed a ubiquitous presence of EBV in the tumor. **B** LMP-1 was immunohistochemically expressed only the in the p63 and p40- positive domain of the tumor. **C** This area revealed a strong and diffuse positivity for p16. **D** No presence of high-risk HPV viruses in this tumor area in the RNA in situ hybridization. **E** and **F** Wildtype expression pattern of p53 and Rb1. **G** The jigsaw puzzle-like domain of the tumor exhibited a moderate complete membranous HER2 expression. **H** No amplification of *ERBB2* was detected in the fluorescence in situ hybridization, Scale bar 20 μm. **I** Intense membranous positivity SSTR2 in both tumor regions. Scale bar for ISH and IHC 200 μm, insets 20 μm

Macroscopically, we observed a 23 × 21 mm unencapsulated tumor, with a firm texture and a lobulated, tan-white cut surface. The tumor exhibited a nodular growth infiltrating the periglandular adipose tissue and displayed a well demarcated bi-morphic histological pattern (Fig. 1C). Approximately two thirds of the tumor was organized into confluent cords separated by lymphoid stroma, creating a jigsaw puzzle-like appearance (Fig. 1C and E). The remaining part of the tumor was organized in broader sheets of syncytial neoplastic cells with scarce infiltrating lymphocytes (Fig. 1C and D). Lymphocytic sialadenitis was observed on the tumor margins, whereas no granulomatous inflammation was noted. The syncytial-appearing neoplastic cells with ill-defined borders had large nuclei with a vesicular chromatin and prominent eosinophilic nucleoli (Fig. 1D and E). Both tumor domains showed increased number of mitoses (> 5 mitoses/1mm^2^). Immunohistochemically, the tumor cells showed “meshwork” pattern of cytokeratin (CK) 5/6 in the jigsaw puzzle-like domain of the tumor and a rather patchy pattern in the other tumor domain (Fig. 1F and G). The latter region strongly expressed p63 and p40, while the former displayed scarce and irregular nuclear positivity for p63 only (Fig. 1H and J). In situ hybridization for EBER, non-coding small RNAs of EBV, revealed a diffuse and ubiquitous nuclear positivity, confirming transcriptionally active EBV in this tumor (Fig. 2A). The p63- and p40- positive regions displayed a membranous and cytoplasmic granular positivity for LMP-1, while the other tumor region did not (Fig. 2B). Neither region showed nuclear positivity for EBNA2 (data not shown). Strikingly, the LMP-1-positive tumor region showed strong, diffuse nuclear and cytoplasmic positivity for p16 (Fig. 2C). No HPV co-infection was detected via in situ hybridization of the oncogenic viral mRNAs E6 and E7 of 18 high-risk HPV subtypes (Fig. 2D). Likewise, a PCR-based assay comprising 28 subtypes of high and low-risk HPV genotypes also failed to reveal any evidence of HPV co-infection (s. Methods). Both Retinoblastoma 1 (Rb1) and p53 immunohistochemistry showed preserved and wildtype expression patterns, respectively (Fig. 2E and F).

The jigsaw puzzle-like domain, on the other hand, exhibited a moderate, complete membranous positivity for HER2 in ca. 60% of tumor cells (Fig. 2G), analogous to a 2+ (equivocal) HER2 expression score described in breast cancer [12]. A subsequent fluorescent in situ hybridization analysis failed to detect an amplification of the *ERBB2* gene either within the HER2-expressing or the HER2-negative tumor region (Fig. 2H). Finally, both tumor regions exhibited strong membranous reactivity for the G-protein-coupled receptor, somatostatin receptor 2 (SSTR2) in > 50% tumor cells (Fig. 2I), analogous to score 3 described in neuroendocrine tumors [13].

To rule out a concurring tumor, we performed the recently established RNA-based next generation sequencing assay involving 27 genes implicated in salivary gland neoplasms (“SalvGlandDx” panel; [11]). The assay was performed separately in both tumor domains and did not reveal any mutation, fusion or overexpression of these genes, further pointing to EBV as a primary cause of this tumor. Finally, the tumor cells did not express CD117, S100, androgen receptor or DOG-1 (data not shown).

## Discussion

Here we describe an EBV-associated lymphoepithelial carcinoma of the parotid gland with a unique and strictly demarcated bi-morphic histological profile and mutually exclusive immunohistochemical expression of p16 and HER2. To the best of our knowledge, this is the first case of an EBV-associated lymphoepithelial carcinoma of the salivary glands that simultaneously expresses these two proteins. Moreover, the tumor also expressed SSTR2, further extending the monitoring and therapeutic window of opportunities for EBV-associated LESCG.

p16 expression was observed in the tumor domain consisting of solid sheets of large neoplastic cells with ill-defined borders, resembling the undifferentiated non-keratinizing nasopharyngeal carcinoma (the so-called “Schmincke pattern” [14], [15]). This tumor region also displayed a strong and diffuse nuclear expression of p63 and p40. Both in situ hybridization and PCR analyses failed to detect any of the commonly tested HPV strains. This is consistent with previously reported cases of both sinonasal and LESCG of the salivary glands [16]. In contrast, a few cases of non-keratinizing nasopharyngeal carcinoma with an EBV and HPV co-infection have previously been reported [7], reviewed in [8]. Conversely, HPV-presence has been demonstrated in a fraction of the laryngeal and hypopharyngeal lymphoepithelial carcinoma while the majority of them lack an EBV-infection [17].

An HPV-independent p16 overexpressing LESCG in the presence of LMP-1, such as the one described here, contradicts previously published studies. Namely, in vitro studies involving human fibroblasts in the early 2000s showed that LMP-1 downregulates p16 by blocking its transcription factor Ets2 [18]. Consistent with that, EBV-associated NPC and gastric carcinoma exhibited homozygous deletions of *CDKN2A* or silenced p16 via hypermethylation of the *CDKN2A* gene promoter region, respectively [19], [20]. p16 overexpression has been linked to cell senescence, and it has been reported that LMP-1 counteracts p16 expression [21], [22]. The p16 overexpression identified here could thus be interpreted as LMP-1 antisenescence insufficiency; however, the high mitotic activity and proliferation index (Ki-67 > 70%; data not shown) observed in this area argues against a state of tumor senescence in this tumor region. We also did not observe a loss of Rb1 expression which could explain p16 overexpression [23].

On the other hand, larger tumor areas displayed anastomosing islands of neoplastic cells with well-defined cell borders and lymphoid stroma, resembling a differentiated non-keratinizing nasopharyngeal carcinoma (the so-called “Regaud pattern” [14], [15]). This tumor domain correlated with a “meshwork” pattern of CK 5/6 signal and HER2-overexpression. The aforementioned gastric carcinoma study also showed that 17% of EBV-associated gastric carcinoma exhibit HER2 amplifications [20]. In salivary gland tumors, HER2 overexpression/amplification has hitherto been reported primarily in salivary duct carcinoma [24], as well as in a recently described case of myoepithelial carcinoma [25]. How EBV might induce HER2 overexpression remains unknown. LMP-1 is known to induce HER1 signaling via the NFκB and STAT3 pathways [26], thus playing a crucial role in nasopharyngeal carcinoma tumorigenesis and prognosis [27], [6]). Whether HER2 signaling is induced by alternative pathways, particularly since it does not correlate with LMP-1 expression, remains to be determined. Immunohistochemically, we observed a p53 wildtype expression pattern, which likely excluded a *TP53* mutation as the cause of HER2 overexpression [28], [29]. We did not test for (rare) somatic *ERBB2* mutations, which can also lead to 2+ (equivocal) HER2 positivity [30].

Finally, recent studies have reported SSTR2 expression in > 80% of EBV-associated NPC, and in vitro studies have demonstrated SSTR2 induction by LMP-1 and NFκB signaling [31], [32]. In this case, SSTR2 was expressed in both immunohistochemically positive and negative LMP-1 tumor regions. We did not test for the activation of the NFκB signaling pathway.

In summary, this case suggests that EBV-driven tumorigenesis in the salivary gland does not involve random genomic aberrations but rather specific activation of diverse growth pathways. The largely atypical immunophenotype (summarized in Table 1) of this case highlights once more the diagnostic challenges that lymphoepithelial carcinoma still poses. Apart from the mutually exclusive p16 and HER2 expression of this tumor, we also observed an inconsistent cytoplasmic CK5/6 positivity within the tumor and large areas of p63 and p40 negativity - all potential diagnostic pitfalls.

**Table 1:** A summary of the heterogenous immunophenotype of the tumor

Marker	Sheets of syncytial cells domain	Jigsaw puzzle-like domain
EBER	Diffuse nuclear positivity	Diffuse nuclear positivity
HER2	Negative	Complete membranous
CK 5/6	Patchy positivity	Meshwork pattern
p16	Diffuse block-type positivity	Negative
LMP-1	Cytoplasmic positivity	Negative
p40	Diffuse positivity	Negative
p63	Diffuse positivity	Focally positive
SSTR2	Strong membranous positivity	Strong membranous positivity
p53	Heterogeneous nuclear positivity	Heterogeneous nuclear positivity
Rb1	Diffuse nuclear positivity	Diffuse nuclear positivity
EBNA2	Negative	Negative
